# Body mass index throughout adulthood, physical activity, and risk of multiple myeloma: a prospective analysis in three large cohorts

**DOI:** 10.1038/s41416-018-0010-4

**Published:** 2018-03-12

**Authors:** Catherine R. Marinac, Brenda M. Birmann, I-Min Lee, Bernard A. Rosner, Mary K. Townsend, Edward Giovannucci, Timothy R. Rebbeck, Julie E. Buring, Graham A. Colditz

**Affiliations:** 1 Department of Medical Oncology, Dana-Farber Cancer Institute, Boston, MA 02115 USA; 2 Department of Epidemiology, Harvard T.H. Chan School of Public Health, Boston, MA 02115 USA; 3 Channing Division of Network Medicine, Department of Medicine, Brigham and Women’s Hospital and Harvard Medical School, Boston, MA 02115 USA; 4 Division of Preventive Medicine, Department of Medicine, Brigham and Women’s Hospital and Harvard Medical School, Boston, MA 02115 USA; 5 Department of Cancer Epidemiology, Moffitt Cancer Center, 12902 Magnolia Drive, Tampa, FL 33612 USA; 6 Department of Nutrition, Harvard School of Public Health, Boston, MA 02115 USA; 7 Department of Surgery and Alvin J. Siteman Cancer Center, Washington University School of Medicine, and Barnes Jewish Hospital, St. Louis, MO 63110 USA

**Keywords:** Risk factors, Cancer epidemiology, Myeloma

## Abstract

**Background:**

Obesity is the only known modifiable multiple myeloma (MM) risk factor. However, the influence of obesity in earlier or later adulthood and the role of other energy balance correlates in MM development are unclear.

**Methods:**

We leveraged repeatedly updated data from the Nurses’ Health Study, Health Professionals Follow-up Study, and Women’s Health Study cohorts to further explore energy balance measures in MM etiology. Exposures derived from questionnaires included young adult body mass index (BMI), cumulative average BMI, BMI change since young adulthood, and cumulative average physical activity and walking. We assessed MM risk related to those variables with Cox proportional hazard models.

**Results:**

We observed 575 incident MM cases in over five million person-years of follow-up across the cohorts. In pooled analyses, MM risk increased 17% per 5 kg/m^2^ increase in cumulative average BMI (95% confidence interval (CI): 1.05, 1.29) and 28% per 5 kg/m^2^ increase in young adult BMI (CI: 1.12, 1.47); adjustment for BMI change since young adulthood did not affect either association. BMI change since young adulthood and cumulative average physical activity and walking were not significantly associated with MM risk.

**Conclusions:**

These findings suggest that a high BMI in early and later adulthood are risk factors for MM.

## Introduction

Multiple myeloma (MM) is an incurable malignancy of plasma cells, characterised by high levels of a monoclonal (“M”) protein in peripheral blood and/or urine, bone marrow plasmacytosis and clinical signs of organ damage.^1^ MM is preceded by an asymptomatic premalignant condition known as monoclonal gammopathy of undetermined significance (MGUS),^2, 3^ and individuals with MGUS have a risk of progression to MM of ~1–2% per year.^4^ MM is expected to account for >32,800 new cancer diagnoses and >12,500 cancer deaths in the United States (US) in 2017.^5^ Five-year relative survival has improved by more than 15 percentage points since the year 2000 due to therapeutic advancements;^6–8^ nonetheless it remains low at 52.7%.^9^ Altogether, these data underscore the urgency of identifying modifiable risk factors for disease incidence, which may be used to inform prevention efforts.

Most established risk factors, including older age, male sex, African ancestry, and family history of haematologic malignancy, are not modifiable. An exception is obesity, which has demonstrated a positive association with MM risk in epidemiologic studies.^10–12^ Indeed, the strength and consistency of the evidence supporting a positive association of body mass index (BMI) with MM led an expert panel convened by the International Agency for Research on Cancer (IARC) to conclude in 2016 that a preventative relationship has been established between “the absence of excess body fatness” and MM.^13^

In light of the established association of obesity with MM, clarification is warranted for other aspects of the relationship, such as whether BMI in both earlier and later life are important. Several published studies suggest that both younger and usual adult BMI are positively associated with MM risk,^10, 14^ including a recent pooled case–control study by the International Multiple Myeloma Consortium (IMMC) with data on both younger and usual adult BMI for 1164 cases and 3629 controls.^10^ Nonetheless, other prospective studies did not observe associations with MM for younger adult BMI,^15, 16^ including an analysis among 121,216 (*n* = 111 MM cases) women enrolled in the California Teachers Study.^16^ It is also unclear whether BMI change or physical activity also influences the risk of MM. The IARC consensus report could not address these questions because the existing evidence is limited (i.e., examined in few and/or under-powered studies) and inconclusive. We undertook the present analysis in the Nurses’ Health Study (NHS), Health Professionals Follow-up Study (HPFS) and Women’s Health Study (WHS) cohorts, with a pooled total of 575 cases of MM and 5,042,395 person-years of follow-up, to elucidate these additional questions and better inform the translation of the current knowledge on obesity to strategies to diminish MM risk. This analysis updates and substantially expands an early report on simple updated (“current”) BMI and cumulative average hours/week of physical activity and MM risk in the NHS and HPFS.^17^ The prior study included 10 fewer years of follow up and only 215 MM cases, and did not include data from WHS participants or examine other anthropometric variables or their changes over time.

## PATIENTS AND Methods

### Study populations

The NHS was established in 1976 with 121,700 female U.S. registered nurses ages 30–55 years who completed the enrollment questionnaire.^18^ The HPFS enrolled 51,529 U.S. licensed male health professionals ages 40–75 years in 1986. Participants in both cohorts have returned follow-up questionnaires biennially since enrollment to update lifestyle and disease history information (http://nurseshealthstudy.org/participants/questionnaires, https://sites.sph.harvard.edu/hpfs/hpfs-questionnaires/). The WHS originally enrolled 39,876 healthy U.S. female health professionals ages 45 years and over in 1992 for a randomised trial of aspirin and vitamin E for cardiovascular disease and cancer prevention.^19^ Since completion of the trial in 2004, women have been followed in an observational study (http://whs.bwh.harvard.edu/). Participants in WHS reported on medical history and lifestyle characteristics at baseline and throughout follow-up by means of questionnaires. HPFS, NHS and WHS participants with a baseline history of cancer other than non-melanoma skin cancer were excluded from the present analyses, leaving a combined baseline total of 49,374 men and 153,260 women.

The protocols for all three cohorts and the present analysis were approved by the Institutional Review Boards at Brigham and Women’s Hospital and the Harvard T.H. Chan School of Public Health. Informed consent was implied by return of the baseline questionnaire (HPFS, NHS) or provided in writing (WHS).

### Assessment of height, weight, and BMI

Participants in each cohort reported their current height and weight on the baseline questionnaires and updated current weight on each follow-up questionnaire. Of note, self-reported and technician-measured weights (r = 0.97) were highly correlated in a subsample of participants.^20^ Body mass index (BMI) for a given follow-up period was calculated as weight (kilograms) divided by height (meters) squared (kg/m^2^).

### Assessment of young adult BMI

HPFS participants reported their weight at age 21 in 1986 (baseline); women in NHS reported their weight at age 18 in 1980, and WHS participants reported their minimum and maximum weight between ages 18–30 years on the questionnaire administered 24-months after baseline. Young adult BMI was calculated using height at enrollment, and using weight at age 21 for HPFS and weight at age 18 for NHS participants. In a similarly-designed companion cohort of women ages 25–42, self-recalled weight at age 18 was strongly correlated (*r* = 0.87) with measured weight in medical records.^21^ In WHS, young adult BMI was approximated using the average of the minimum and maximum weight between the ages of 18 and 30.

### Assessment of physical activity

Detailed information on recreational physical activity (walking, running, jogging, bicycling, racquet sports, calisthenics/aerobics, swimming, weight training etc.) during the past year was assessed in each study by standardised questionnaires--beginning in 1986 in HPFS and NHS and at baseline in WHS, and typically updated every 2–4 years during follow-up. To incorporate activity frequency, duration, and intensity into a summary measure of energy expenditure, we calculated total metabolic equivalent (MET) hours of all activity and of walking per week.^22^ These physical activity measures have also been validated in both men and women.^23, 24^

### Outcome ascertainment

The outcome of interest was first primary diagnosis of MM, which we identified primarily through self-report on the follow-up questionnaires, with additional cases identified when confirming vital status. Deaths were identified by next-of-kin, the postal service or routine searches of the National Death Index, which was shown to be highly sensitive and specific in these cohorts.^25, 26^ To confirm cases, we sought written consent for medical record review from the participant or from next of kin (if deceased); trained personnel reviewed medical records to confirm the occurrence and date of MM diagnosis. When the original medical records were unavailable, we sought to confirm case diagnoses via linkage to state tumour registries. Follow-up time was censored in 2012 for NHS and HPFS and in the third observational follow-up period for WHS, which was in 2008.

### Statistical analyses

Person-time was calculated from study enrollment to the earliest among dates of diagnosis of MM, another cancer (except non-melanoma skin cancer), death, or the end of follow-up. For analyses of physical activity and walking in NHS, follow-up began in 1986. The exposures of interest included cumulative average BMI, young adult BMI, change in BMI since young adulthood, cumulative average physical activity, and cumulative average walking. We calculated cumulative average variables as the mean of all available information from baseline through each new follow-up cycle, to better reflect individuals’ long-term exposure and diminish the influence of misclassification in any given follow-up cycle.^27^ Cumulative average BMI was categorised as <23 kg/m^2^, 23–<25 kg/m^2^, 25–<27.5, 27.5–<30 kg/m^2^, and ≥30 kg/m^2^, consistent with World Health Organization (WHO) categories^28^ except that the usual “normal BMI” category of <25 kg/m^2^ group, and “overweight BMI” category of 25–<30, which were sizable in these cohorts, were split to permit a finer assessment of dose-response and to mirror the previously published study of BMI and MM risk in NHS and HPFS.^17, 29^ Due to limited reports of obesity in young adulthood, we modeled only three categories of young adult BMI (<23 kg/m^2^, 23–<25 kg/m^2^, ≥25 kg/m^2^). Furthermore, BMIs considered underweight (<18.5 kg/m^2^) were uncommon and thus excluded from all models that included younger or usual adult (i.e., cumulative average) BMI. Change in BMI since young adulthood (<0 kg/m^2^, 0–1.5 kg/m^2^, >1.5–3 kg/m^2^, >3 kg/m^2^) was modeled with simple updates at each follow-up cycle. Cumulative average physical activity was categorised as 0–<9, 9–<18, 18–<27, ≥27 MET-hours/week and cumulative average walking as 0–<3, 3–<9, 9–<18, and ≥18 MET-hours/week. We also categorised physical activity according to adherence to the World Cancer Research Fund and American Institute for Cancer Research (WCRF/AICR) joint physical activity recommendations, which we operationalised as ≥210 min per week.^30^ Finally, for comparison with corresponding BMI variables, we also examined height, current weight, young adult weight and change in weight since young adulthood in relation to MM risk. We categorised height and (biennially updated) weight variables in quartiles defined in non-cases and calculated updated change in weight since young adulthood (≤5, >5–10, >10–20, >20 kg). To test for linear trend, we further examined each exposure as a continuous variable, specifically in 5 kg/m^2^ increments of cumulative average and young adult BMI and change in BMI; in 5 kg increments for weight and weight change variables; and in 10 MET-hour/week increments for the physical activity variables. A one-cycle carry forward rule was applied to variables to minimise missing data.

We examined interrelationships between young adult and adult BMI variables with Spearman correlations. In separate Cox proportional hazards models stratified by age (months) and calendar period of follow-up, we calculated hazards ratios (HRs) and 95% confidence intervals (CI) for the association of a given energy balance exposure category or continuous measure with the risk of MM. To avoid potential collinearity when evaluating whether young adult and usual adult BMI associations with MM were independent of each other, we ran additional models of each BMI variable with adjustment for change in BMI since young adulthood; we also examined interactions between young adult and usual adult BMI in a model that included both main effects and a cross-product term, and examined joint associations of young adult and cumulative average BMI. For consistency across modeling strategies, and to examine whether associations observed were independent of BMI earlier in life, models of change in BMI since young adulthood were also run with and without adjustment for young adult BMI. Models of cumulative average physical activity and walking were run with and without adjustment for cumulative average BMI; and all models of weight and weight change additionally controlled for height.

Statistical outlier values were identified using the Rosner extreme studentised deviate method,^31^ and all main models were re-run without exposure outlier values as a sensitivity analysis. To assess the potential influence of weight loss due to subclinical MM, we also implemented a two-cycle (i.e., 4-year) exposure lag and examined the lagged exposures in sensitivity analyses that utilised a similar series of Cox models to those previously described. Tests of interactions between the exposure of interest and the (log-)time scale verified the proportional hazards assumption in all main models. After pooling NHS and WHS data by random-effects meta-analysis, final models were run separately by sex, and a random-effects meta-analysis was used to test for heterogeneity by sex. We also derived “pooled” summary effect estimates across the three cohorts using a random-effects meta-analysis and tested for heterogeneity by cohort.

## Results

We confirmed incident diagnoses of MM in 205 men (HPFS) and 370 women (325 NHS, 45 WHS). Participants were of similar average age in all three cohorts and were predominantly White (Table 1). Mean BMI ranged from 23.8–25.8 kg/m^2^ at baseline, and from 21.3–23.0 kg/m^2^ in young adulthood.

**Table 1:** Characteristics of the Health Professionals Follow-Up Study (HPFS), Nurses’ Health Study (NHS), and Women’s Health Study (WHS)^a^

	HPFS (*n* = 49,374)	NHS (*n* = 118,230)	WHS (*n* = 35,030)
Age at enrollment, years	54.50 (9.8)	48.82 (7.2)	54.35 (6.9)
Age at end of follow-up, years	73.63 (7.9)	75.29 (6.9)	67.66 (6.8)
*Race*			
White, %	91	97	94
Black, %	1	2	2
Asian, %	2	1	1
Other/miss, %	7	0	2
Body mass index, kg/m²	25.54 (3.4)	23.76 (4.2)	25.82 (5.0)
Young adult body mass index, kg/m²	23.02 (3.0)	21.34 (3.0)	21.87 (3.1)
Change in BMI since young adulthood, kg/m²	2.52 (2.9)	2.39 (3.6)	3.92 (3.8)
Activity, MET-h/week^b^	18.75 (26.3)	14.07 (21.0)	14.58 (18.3)
Walking, MET-h/week^c^	8.30 (13.6)	6.53 (9.4)	7.28 (7.2)

Values of polytomous variables may not sum to 100% due to rounding

^a^Values are mean (SD) and ascertained at baseline unless otherwise noted

^b^Metabolic equivalent hours per week from recreational and leisure time activities

^c^Metabolic equivalent hours per week from recreational and leisure time activities (walking only)

We noted weak to moderate correlations for young adult and later adult BMI variables in each cohort (Supplementary Table 1). The correlations with young adult BMI were slightly stronger for baseline BMI than for cumulative average BMI and somewhat weaker for current BMI and change in BMI since young adulthood.

We observed a positive association with MM risk for both cumulative average adult BMI and young adult BMI (Table 2). Although the associations were somewhat stronger among men, there was no statistically significant heterogeneity in the sex- or cohort-specific effect estimates (all *p*-values for heterogeneity ≥0.20 and ≥0.42, respectively), so we focus herein on the pooled (e.g., meta-analysed) results across all cohorts. Accordingly, in pooled analyses, each 5 kg/m^2^ increase in cumulative average adult BMI was associated with a statistically significant 17% increased risk of MM (HR: 1.17; 95% CI: 1.05, 1.29). Adjustment for change in BMI since young adulthood had only minimal influence (Table 2). MM risk also increased by nearly 30% per 5 kg/m^2^ increase in young adult BMI (HR: 1.28; 95% CI: 1.12, 1.47), with no appreciable effect of adjustment for change in BMI since young adulthood. We did not see evidence of statistical interaction between BMI in young adulthood and usual adult BMI (cohort-specific *P*-values for interaction all ≥0.32), or trends across joint categories of cumulative average and young adult BMI (Supplement Table 2). Change in BMI since young adulthood was suggestively but not significantly associated with MM risk (*p* = 0.10), with adjustment for young adult BMI.

**Table 2:** BMI and the risk of incident multiple myeloma in men and women

	Men			Women^a^			Pooled^b^
	Cases	Person-years	HR (95% CI)	Cases	Person-years	HR (95% CI)	HR (95% CI)
*Cumulative average BMI* *(kg/m* ^*2*^ *)*							
18.5–<23 kg/m^2^	20	143351	1.00	88	1218619	1.00	1.00
23–<25 kg/m^2^	54	238232	1.77 (1.04, 3.00)	61	669099	0.99 (0.61, 1.61)	1.19 (0.76, 1.88)
25–<27.5 kg/m^2^	52	265658	1.54 (0.90, 2.63)	56	571908	1.00 (0.65, 1.52)	1.14 (0.79, 1.65)
27.5–<30 kg/m^2^	22	126896	1.41 (0.76, 2.63)	53	333742	1.63 (1.16, 2.30)	1.44 (0.99, 2.08)
≥30 kg/m^2^	21	78753	2.27 (1.21, 4.28)	44	419586	1.14 (0.79, 1.65)	1.38 (0.83, 2.27)
HR per 5 kg/m^2^			1.25 (1.03, 1.52)			1.14 (1.01, 1.28)	1.17 (1.05, 1.29)
*P*_trend_			0.02			0.04	0.003
*Cumulative average BMI* *(kg/m* ^*2*^ *)* *adjusted for* *change in BMI since young adulthood*							
18.5–<23 kg/m^2^	20	143351	1.00	88	1218619	1.00	1.00
23–<25 kg/m^2^	54	238232	1.82 (1.07, 3.09)	61	669099	1.02 (0.73, 1.43)	1.20 (0.75, 1.91)
25–<27.5 kg/m^2^	52	265658	1.65 (0.96, 2.85)	56	571908	0.98 (0.68, 1.41)	1.15 (0.77, 1.71)
27.5–<30 kg/m^2^	22	126896	1.57 (0.82, 3.00)	53	333742	1.55 (1.05, 2.29)	1.55 (1.11, 2.17)
≥30 kg/m^2^	21	78753	2.70 (1.35, 5.42)	44	419586	1.05 (0.66, 1.67)	1.48 (0.75, 2.90)
HR per 5 kg/m^2^			1.33 (1.09, 1.63)			1.13 (0.95, 1.33)	1.20 (1.06, 1.37)
*P*_trend_			0.006			0.16	0.005
*Young adult BMI* *(kg/m* ^*2*^ *)*							
18.5–<23 kg/m^2^	69	403041	1.00	195	2153960	1.00	1.00
23–<25 kg/m^2^	49	230418	1.37 (0.94, 1.99)	39	396692	1.29 (0.59, 2.85)	1.27 (0.85, 1.89)
≥25 kg/m^2^	43	194116	1.49 (1.01, 2.19)	31	321214	1.11 (0.76, 1.62)	1.28 (0.97, 1.68)
HR per 5 kg/m^2^			1.39 (1.16, 1.67)			1.17 (0.95, 1.42)	1.28 (1.12, 1.47)
*P*_trend_			0.0004			0.13	0.0003
*Young adult BMI adjusted for change in BMI since young adulthood*							
18.5–<23 kg/m^2^	69	403106	1.00	195	2153960	1.00	1.00
23–<25 kg/m^2^	49	230418	1.36 (0.93, 1.99)	39	396692	1.30 (0.60, 2.85)	1.27 (0.86, 1.89)
≥25 kg/m^2^	43	194116	1.48 (0.99, 2.21)	31	321214	1.14 (0.78, 1.68)	1.29 (0.98, 1.70)
HR per 5 kg/m^2^			1.42 (1.16, 1.73)			1.19 (0.97, 1.46)	1.30 (1.13, 1.50)
*P*_trend_			0.0006			0.09	0.0003
*Change in BMI since young adulthood adjusted for young adult BMI*							
<0 kg/m^2^	23	109500	0.75 (0.43, 1.30)	30	393638	0.86 (0.52, 1.44)	0.81 (0.55, 1.17)
0–1.5 kg/m^2^	34	180818	1.00	32	423290	1.00	1.00
>1.5–3.0 kg/m^2^	36	181252	1.03 (0.64, 1.66)	38	462122	1.07 (0.66, 1.71)	1.05 (0.75, 1.47)
>3 kg/m^2^	68	356004	1.00 (0.65, 1.52)	165	1592817	1.10 (0.75, 1.62)	1.05 (0.79, 1.40)
HR per 5 kg/m^2^			1.06 (0.85, 1.32)			1.10 (0.98, 1.24)	1.09 (0.98, 1.21)
*P*_trend_			0.63			0.10	0.10

*P*-values of tests for heterogeneity by sex were all *P* ≥ 0.20; *P*-values of tests for heterogeneity by cohort were all *p* ≥ 0.42. Cohort-specific data are available from the authors upon request

*P*-values for trend tests are derived from models with the exposure of interest modeled as a continuous variable

*BMI* body mass index

^a^Data were pooled across the women-only cohorts using a random-effects meta-analysis

^b^Data were pooled across the three cohorts using a random-effects meta-analysis

Models of weight in adulthood, young adulthood, and weight change were directionally similar to the models of BMI described above (and *P*-values for heterogeneity by sex and cohort were all ≥0.22 and ≥0.39, respectively), although the HR for weight change since young adulthood reached statistical significance (Supplement Table 3). Each 5 kg increase in weight change since young adulthood was associated with a significant 4% increased risk of developing MM (pooled HR: 1.04; 95% CI: 1.00, 1.08; *P* = 0.03) when adjusted for height and young adult weight. We did not observe statistically significant associations with MM risk for adult height (Supplement Table 3), nor did we observe statistically significant associations with MM risk for any physical activity variables (Table 3) or evidence of interaction of a physical activity variable with BMI in relation to MM risk (cohort-specific *P*-values for interaction all ≥0.40). Sensitivity analyses confirmed that the outlier records (*N* = 2196 for cumulative average BMI; *N* = 3366 for young adult BMI; *N* = 9160 for physical activity; *N* = 6935 for walking) and a 4-year exposure lag did not influence the main findings.

**Table 3:** Physical activity, walking, and the risk of incident multiple myeloma in men and women

	Men			Women^a^			Pooled^b^
	Cases	Person-years	HR (95% CI)	Cases	Person-years	HR (95% CI)	HR (95% CI)
*Cumulative average physical activity (MET-hours/week)*							
0–<9 MET-hrs	41	234922	1.00	134	1060299	1.00	1.00
9–<18 MET-hrs	40	208615	1.04 (0.67, 1.62)	86	666960	0.93 (0.70, 1.22)	0.96 (0.76, 1.21)
18–<27 MET-hrs	36	163131	1.17 (0.74, 1.85)	37	370575	0.70 (0.48, 1.00)	0.84 (0.55, 1.27)
≥27 MET-hrs	85	361618	1.33 (0.90, 1.96)	51	440449	0.85 (0.61, 1.18)	1.03 (0.73, 1.45)
HR per 10 MET-hrs			1.03 (0.97, 1.09)			0.94 (0.87, 1.02)	0.98 (0.92, 1.06)
*P*_trend_			0.42			0.11	0.66
*Cumulative average physical activity (MET-hours/week), adjusted for cumulative average BMI*							
0–<9 MET-hrs	41	234922	1.00	134	1060299	1.00	1.00
9–<18 MET-hrs	40	208615	1.06 (0.68, 1.65)	86	666960	0.94 (0.72, 1.25)	0.98 (0.77, 1.23)
18–<27 MET-hrs	36	163131	1.20 (0.75, 1.90)	37	370575	0.72 (0.50, 1.04)	0.86 (0.56, 1.31)
≥27 MET-hrs	85	361618	1.40 (0.94, 2.07)	51	440449	0.88 (0.63, 1.23)	1.07 (0.76, 1.51)
HR per 10 MET-hrs			1.03 (0.97, 1.10)			0.95 (0.88, 1.02)	0.99 (0.93, 1.06)
*P*_trend_			0.27			0.18	0.84
*Cumulative average walking (MET-hours/week)*							
0–<3 MET-hrs	47	279895	1.00	101	794822	1.00	1.00
3–<9 MET-hrs	69	329316	1.09 (0.75, 1.59)	126	948009	1.19 (0.55, 2.58)	1.04 (0.77, 1.41)
9–<18 MET-hrs	43	205327	0.93 (0.61, 1.43)	51	512466	1.14 (0.27, 4.85)	0.96 (0.51, 1.82)
≥18 MET-hrs	43	153730	1.22 (0.79, 1.86)	25	232783	0.79 (0.51, 1.23)	0.99 (0.72, 1.35)
HR per 10 MET-hrs			1.05 (0.92, 1.18)			0.93 (0.79, 1.09)	1.00 (0.91, 1.10)
*P*_trend_			0.48			0.35	0.97
*Cumulative average walking (MET-hours/week), adjusted for cumulative average BMI*							
0–<3 MET-hrs	47	279895	1.00	101	794822	1.00	1.00
3–<9 MET-hrs	69	329316	1.11 (0.76, 1.62)	126	948009	1.20 (0.57, 2.51)	1.06 (0.79, 1.41)
9–<18 MET-hrs	43	205327	0.95 (0.62, 1.46)	51	512466	1.15 (0.28, 4.68)	0.98 (0.53, 1.80)
≥18 MET-hrs	43	153730	1.25 (0.81, 1.92)	25	232783	0.83 (0.53, 1.30)	1.03 (0.76, 1.40)
HR per 10 MET-hrs			1.05 (0.93, 1.19)			0.95 (0.81, 1.11)	1.01 (0.92, 1.12)
*P*_trend_			0.42			0.51	0.82

*P*-values of tests for heterogeneity by sex were all *P* ≥ 0.08; *P*-values of tests for heterogeneity by cohort were all *P* ≥ 0.19. Cohort-specific data are available from the authors upon request

*P*-values for trend tests are derived from models with the exposure of interest modeled as a continuous variable.

*BMI* body mass index, *hrs* hours, *MET* Metabolic Equivalent

^a^Data were pooled across the women-only cohorts using a random-effects meta-analysis

^b^Data were pooled across the three cohorts using a random-effects meta-analysis

## Discussion

In this analysis of data from three large prospective cohorts, a higher BMI in both later adulthood and young adulthood was associated with a similarly increased risk of MM. This association did not significantly differ by gender but was nonetheless slightly stronger in men. MM risk was significantly positively associated with weight change and suggestive of a positive association for change in BMI since young adulthood. In contrast, we did not observe statistically significant associations of cumulative average physical activity or walking with MM risk.

In addition to supporting the strong evidence base for a causal association between obesity and MM risk,^13^ the current study addresses important knowledge gaps surrounding the importance of BMI in earlier and later life for MM risk, a question investigated in only a few studies. An analysis in the IMMC (1164 cases and 3629 controls with both younger and usual adult data) reported a strong statistical interaction between younger and usual adult BMI, with joint models indicating that MM risk was strongest among individuals who were obese at both times, compared to those whose BMI was normal at both times.^10^ Another joint analysis conducted in an even larger prospective pooled study (20 cohorts, 1.5 million participants, 1388 MM deaths) observed the highest MM mortality in women with a BMI ≥ 30 both in young adulthood and at baseline but no apparent joint effect in men.^32^ A third prospective study reported similar positive associations of MM risk with BMI in young adulthood and adulthood, with no significant interaction.^14^ The current study supports the latter findings that BMI in younger and later adulthood are both important for MM risk but did not replicate a statistical interaction or trends across jointly classified categories of the two BMI variables. However, it is notable that our case count accommodated only two categories of each BMI variable in contrast to the three finer categories utilised in the IMMC analysis. Other published studies did not observe an association with MM for younger adult BMI, including both case–control and prospective studies limited by small numbers of MM cases and/or insufficient variability of young adult BMI to examine overweight or obese values.^15, 16, 33, 34^ Collectively, the present and previous evidence from the better-powered studies supports an inference that weight control throughout adulthood may confer a benefit of reducing MM risk.

The IARC consensus report found compelling evidence that an absence of excess body fatness in adulthood has a preventative association with MM and asserted that mechanistic evidence supports a causal cancer-preventive effect of weight loss on most cancers.^13^ To date there is little data on MM risk in relation to weight and/or BMI changes in adulthood, a question that may be particularly relevant to MGUS patients given the lack of strategies for minimising progression to malignancy.^35^ We are aware of one previous study of weight change and MM. That study investigated intentional weight loss and lymphohaematopoietic cancer risk among women and found no association between net weight loss since age 35 and MM risk, but had only 92 MM cases and thus had limited statistical power to detect an association.^15^ Our findings, which did not distinguish intentional from unintentional causes, suggest that irrespective of starting BMI in young adulthood, individuals who subsequently reduced their BMI or weight may have had a decreased risk of MM. These data, combined with the mechanistic evidence outlined in the IARC report, suggest that weight loss may confer an added benefit for MM prevention. Studies are warranted to assess whether inclusion of young adult and/or usual adult BMI would improve on current MGUS risk stratification, which is presently based on clinical parameters,^36^ as well as to investigate the influence of weight loss on risk of progression in adults with MGUS that have a high BMI.^37^

Additional evidence supports the plausibility of an obesity-related increase in MM risk; specifically there is strong evidence that physiological dysfunction of adipose tissue in obese persons can promote MM pathogenesis. For example, adipose tissue in obese individuals produces altered concentrations of pro-inflammatory cytokines, lipid metabolites and lypolytic enzymes, as well as altered adipokines and growth factors which can influence the bone marrow microenvironment through systemic signalling pathways.^38–40^ Collectively, along with obesity-associated systemic changes, adipocyte-derived compounds may serve as a fuel source for MM tumour cells and promote their proliferation^41^ and reduce apoptosis.^42, 43^ In support of these putative mechanisms linking obesity with MM development, prospective epidemiologic studies have found associations with future MM incidence for pre-diagnosis concentrations of serologic biomarkers of obesity-related hormonal deregulation thought to contribute to MM pathogenesis.^44, 45^

Physical activity and weight control have downstream effects on many of the same metabolic systems postulated to contribute to myelomagenesis,^46^ and animal data suggest that physical activity may promote a bone marrow microenvironment that is less conducive to tumour initiation.^38, 47^ Therefore, it is intriguing that we did not observe an association between physical activity and MM risk. This lack of association between physical activity and MM risk is consistent with some (but not all) published reports (reviewed in Jochem et al.^48^); most studies reported null or suggestive inverse associations, including one large prospective investigation which found no association between total or leisure-time physical activity and MM risk, and no interaction of physical activity with BMI.^14^ It is noteworthy, however, that to our knowledge, all studies to date^48^ including the present study have used self-report to characterise physical activity, an approach that is prone to measurement error.

Limitations of the present study include the reliance on self-reported measures of weight, height, and physical activity, which although validated, may introduce random error and exposure non-differential misclassification. Also, the current study populations are homogeneous with respect to sociodemographic factors, and therefore, caution must be applied when generalising our findings to more diverse populations. The reliance on an average of minimum and maximum weight from ages 18 to 30 years when computing young adult BMI in the WHS may have introduced misclassification; however, the mean and SD for young adult BMI and correlations among BMI variables were similar across the cohorts, providing reassurance that any such misclassification for WHS participants was minimal. Finally, although there are few established risk factors for MM, and we adjusted for age and studied a predominantly white population, we were not able to adjust for family history of haematologic malignancy or MGUS status and cannot rule out residual confounding by these or other unmeasured risk factors.

Strengths of the present analysis include the prospective design with a relatively large sample size for a prospective study of MM. In addition, the time-varying analysis of cumulative average measures of adult BMI and physical activity levels diminished the influence of misclassification in any given follow-up cycle^27^ and better captured individuals’ longer-term exposure. Furthermore, the assessment of BMI in young adulthood permitted exploration of the relevant timing of exposure in relation to MM risk.

In conclusion, our findings support the growing body of literature demonstrating that a high BMI both early and later in adulthood is associated with the risk of MM, and suggest that maintaining a healthy body weight throughout life may be an important component to a much-needed MM prevention strategy. Further larger-scale studies aimed at clarifying the influence of obesity timing and duration and at directly evaluating the role of weight loss, ideally conducted in diverse prospective study populations and in MGUS patients, will be important for elaborating the role of weight maintenance in MM prevention and for identifying high risk subgroups of patients that may benefit from weight loss.

## Electronic supplementary material

Supplementary tables
