# Supplementary material for: Body mass index throughout adulthood, physical activity, and risk of multiple myeloma: a prospective analysis in three large cohorts
Source: Br J Cancer. 2018 Mar 12;118(7):1013–9. doi: 10.1038/s41416-018-0010-4 (PMC5931105; doi:10.1038/s41416-018-0010-4)
Supplement: Supplementary file 1 — Supplementary tables [file 41416_2018_10_MOESM1_ESM.docx]

| **Supplement Table 1. Spearman Correlations of Adult BMI Variables with Young Adult BMI Among Participants in the Health Professionals Follow-Up Study (HPFS), Nurses’ Health Study (NHS), and Women’s Health Study (WHS).^a^** | | | |
| --- | --- | --- | --- |
|  | **NHS** | **HPFS** | **WHS** |
| **Current BMI** | 0·39 | 0·49 | 0·55 |
| **Cumulative Average BMI** | 0·46 | 0·54 | 0·59 |
| **Baseline BMI** | 0·49 | 0·55 | 0·60 |
| **BMI Change Since Young Adulthood** | -0·17 | -0·30 | 0·02 |
| Abbreviations: BMI=body mass index. | | | |
| ^a^ All the pairwise correlations had p-values <0·001 | | | |

| **Supplement Table 2. Joint Classification of Young Adult and Cumulative Average Body Mass Index (BMI) and the Risk of Incident Multiple Myeloma in Men and Women.** | | | | | | | |  |
| --- | --- | --- | --- | --- | --- | --- | --- | --- |
|  | **Men** | | | **Women^a^** | | | **Pooled^b^** | |
|  | **Cases** | **Person-Years** | **HR (95% CI)** | **Cases** | **Person-Years** | **HR (95% CI)** | **HR (95% CI)** | |
| **Joint Classification of Young Adult (YA) and Cumulative Average (CA) BMI** | | | | | | | | |
| Both <25 | 72 | 363010 | 1·00 | 118 | 1576595 | 1·00 | 1·00 | |
| YA <25, CA≥25 | 61 | 325879 | 0·94 (0·67, 1·33) | 118 | 1035752 | 1·23 (0·95, 1·59) | 1·12 (0·91, 1·38) | |
| YA ≥25, CA<25 | 9 | 28612 | 1·50 (0·74, 3·03) | 4 | 64794 | 1·34 (0·28, 6·34) | 1·35 (0·73, 2·49) | |
| Both ≥25 | 41 | 185447 | 1·27 (0·86, 1·88) | 27 | 267749 | 1·24 (0·82, 1·90) | 1·26 (0·95, 1·68) | |
| P-values of tests for heterogeneity by sex were all P>0·23; P-values of tests for heterogeneity by cohort were all p>0·35. Cohort-specific data are available from the authors upon request.  ^a^Data were pooled across the women-only cohorts using a random-effects meta-analysis.  ^b^Data were pooled across the three cohorts using a random-effects meta-analysis. | | | | | | | |  |

| **Supplement Table 3. Quartiles of Adult Height and Weight and the Risk of Incident Multiple Myeloma in Men and Women.** | | | | | | | |
| --- | --- | --- | --- | --- | --- | --- | --- |
|  | **Men** | | | **Women^a^** | | | **Pooled^b^** |
|  | **Cases** | **Person-Years** | **HR (95% CI)** | **Cases** | **Person-Years** | **HR (95% CI)** | **HR (95% CI)** |
| **Adult Height** | | | | | | | |
| Q1 | 58 | 252988 | 1·00 | 121 | 1409035 | 1·00 | 1·00 |
| Q2 | 63 | 281013 | 1·12 (0·78, 1·62) | 70 | 755350 | 1·14 (0·83, 1·57) | 1·14 (0·91, 1·43) |
| Q3 | 51 | 267866 | 1·07 (0·72, 1·57) | 89 | 1070962 | 0·85 (0·38, 1·89) | 1·05 (0·84, 1·32) |
| Q4 | 32 | 172898 | 1·07 (0·69, 1·67) | 89 | 826977 | 1·38 (1·05, 1·82) | 1·29 (1·02, 1·63) |
| HR per 5 cm |  |  | 1·03 (0·93, 1·14) |  |  | 1·08 (0·99, 1·17) | 1·06 (0·99, 1·13) |
| P*_trend_:* |  |  | 0·55 |  |  | 0·07 | 0·07 |
| **Adult Weight** | | | | | | | |
| Q1 | 40 | 211631 | 1·00 | 61 | 881938 | 1·00 | 1·00 |
| Q2 | 49 | 250889 | 1·19 (0·77, 1·84) | 59 | 724404 | 1·09 (0·76, 1·57) | 1·13 (0·85, 1·49) |
| Q3 | 37 | 178449 | 1·37 (0·84, 2·23) | 93 | 802572 | 1·37 (0·98, 1·92) | 1·37 (1·04, 1·81) |
| Q4 | 43 | 213773 | 1·51 (0·92, 2·48) | 90 | 789392 | 1·32 (0·93, 1·88) | 1·38 (1·04, 1·84) |
| HR per 5 kg |  |  | 1·07 (1·01, 1·14) |  |  | 1·04 (1·00, 1·09) | 1·05 (1·02, 1·09) |
| P*_trend_:* |  |  | 0·03 |  |  | 0·03 | 0·003 |
| **Adult Weight Adjusted for Change in Weight Since Young Adulthood** | | | | | | | |
| Q1 | 40 | 211631 | 1·00 | 61 | 881938 | 1·00 | 1·00 |
| Q2 | 49 | 250889 | 1·27 (0·81, 2·00) | 59 | 724404 | 1·07 (0·73, 1·55) | 1·14 (0·86, 1·53) |
| Q3 | 37 | 178449 | 1·53 (0·91, 2·57) | 93 | 802572 | 1·29 (0·89, 1·88) | 1·37 (1·01, 1·86) |
| Q4 | 43 | 213773 | 1·83 (1·02, 3·29) | 90 | 789392 | 1·31 (0·65, 2·65) | 1·46 (0·93, 2·30) |
| HR per 5 kg |  |  | 1·12 (1·04, 1·22) |  |  | 1·05 (0·98, 1·13) | 1·08 (1·03, 1·14) |
| P*_trend_:* |  |  | 0·004 |  |  | 0·15 | 0·003 |
| **Young Adult Weight** | | | | | | | |
| Q1 | 36 | 177313 | 1·00 | 66 | 743916 | 1·00 | 1·00 |
| Q2 | 38 | 216042 | 1·04 (0·65, 1·66) | 82 | 850232 | 1·08 (0·77, 1·50) | 1·06 (0·81, 1·40) |
| Q3 | 56 | 270549 | 1·34 (0·85, 2·12) | 75 | 743768 | 1·15 (0·73, 1·80) | 1·18 (0·90, 1·56) |
| Q4 | 39 | 190838 | 1·49 (0·88, 2·52) | 80 | 860389 | 1·04 (0·73, 1·49) | 1·17 (0·87, 1·57) |
| HR per 5 kg |  |  | 1·10 (1·03, 1·18) |  |  | 1·04 (0·97, 1·11) | 1·07 (1·02, 1·12) |
| P*_trend_:* |  |  | 0·008 |  |  | 0·27 | 0·009 |
| **Young Adult Weight Adjusted for Change in Weight Since Young Adulthood** | | | | | | | |
| Q1 | 36 | 177313 | 1·00 | 66 | 743916 | 1·00 | 1·00 |
| Q2 | 38 | 216042 | 1·06 (0·65, 1·70) | 82 | 850232 | 1·09 (0·78, 1·53) | 1·08 (0·82, 1·42) |
| Q3 | 56 | 270549 | 1·37 (0·86, 2·20) | 75 | 743768 | 1·15 (0·77, 1·73) | 1·21 (0·91, 1·60) |
| Q4 | 39 | 190838 | 1·55 (0·90, 2·67) | 80 | 860389 | 1·09 (0·76, 1·56) | 1·21 (0·90, 1·64) |
| HR per 5 kg |  |  | 1·12 (1·04, 1·22) |  |  | 1·05 (0·98, 1·13) | 1·08 (1·03, 1·14) |
| P*_trend_:* |  |  | 0·004 |  |  | 0·15 | 0·003 |
| **Change in Weight Since Young Adulthood Adjusted for Young Adult Weight** | | | | | | | |
| ≤5 | 61 | 302463 | 1·00 | 75 | 999227 | 1·00 | 1·00 |
| >5 to 10 | 35 | 192268 | 1·04 (0·68, 1·61) | 51 | 614898 | 1·13 (0·79, 1·63) | 1·09 (0·83, 1·44) |
| >10 to 20 | 47 | 243612 | 1·14 (0·76, 1·71) | 99 | 891675 | 1·20 (0·72, 2·02) | 1·24 (0·97, 1·59) |
| >20 | 26 | 116398 | 1·31 (0·81, 2·13) | 78 | 692506 | 1·19 (0·86, 1·66) | 1·23 (0·94, 1·61) |
| HR per 5 kg |  |  | 1·04 (0·97, 1·12) |  |  | 1·04 (1·00, 1·09) | 1·04 (1·00, 1·08) |
| P*_trend_:* |  |  | 0·28 |  |  | 0·06 | 0·03 |
| P-values of tests for heterogeneity by sex were all >0·22; P-values of tests for heterogeneity by cohort were all >0·39; Cohort-specific data are available from the authors upon request.  All models of weight and weight change were adjusted for height at baseline.  P-values for trend tests are derived from models with the exposure of interest modeled as a continuous variable.  Abbreviations: Q=quartile.  ^a^Data were pooled across the women-only cohorts using a random-effects meta-analysis.  ^b^Data were pooled across the three cohorts using a random-effects meta-analysis. | | | | | | | |
